# Pattern of Emergency and Interdepartmental Ophthalmology Referral Calls and Their Impact on Postgraduate Training at a Tertiary Care Hospital in Central India

**DOI:** 10.7759/cureus.93856

**Published:** 2025-10-05

**Authors:** Sunil Verma, Priti Singh, Saroj Gupta, Samendra Karkhur, Parul Mittal, Vidhya Verma

**Affiliations:** 1 Ophthalmology, Shroff Eye Centre, New Delhi, IND; 2 Ophthalmology, All India Institute of Medical Sciences, Bhopal, IND

**Keywords:** emergency ophthalmology referrals, interdepartmental consultation, learning curve, postgraduate training, tertiary care hospital

## Abstract

Purpose: This study aimed to analyze the pattern and nature of emergency and interdepartmental ophthalmology referrals at a tertiary care hospital and assess their impact on the clinical competency and learning curve of postgraduate (PG) residents.

Methods: A prospective observational study was conducted over 18 months at a tertiary care hospital in Central India. A total of 1061 referrals were analyzed regarding demographics, referring departments, reasons for referral, ocular findings, interventions, and PG resident performance. Resident competency was assessed by comparing their evaluations with those of senior residents or consultants over six-month intervals.

Results: The average patient age was 39.76±22.26 years, with a predominance of male patients (n=650, 61.26%). The majority of referral calls were received on weekdays (n=782, 73.7%), particularly during working hours between 8:00 am and 4:00 pm (n=672, 63.34%). The department of medicine (n=377, 35.53%) and the trauma and emergency department (n=251, 23.66%) contributed the majority of referrals.

The most common reason for referral was for aid in diagnosis (n=333, 31.39%). No ocular abnormalities were found in 648 cases (61.07%). Among those with positive findings, the most frequent were ocular trauma (n=148, 13.95%) and diabetic retinopathy (n=83, 7.83%). Interventions were required in 251 cases (23.66%).

Diagnostic agreement between PG residents and seniors improved significantly from 221 cases (62.96%) to 312 cases (91.22%), and the discrepancy rate decreased from 130 cases (37.01%) to 30 cases (8.77%) over an 18-month period.

Conclusion: Interdepartmental ophthalmology referrals are crucial for both clinical care and as a structured training avenue for PG residents. Regular exposure, supervised feedback, and structured evaluation significantly enhance diagnostic proficiency. Formalizing referral training in residency programs can improve clinical outcomes and interdisciplinary collaboration.

## Introduction

Interdepartmental referrals are key to hospital workflow and facilitate collaborative care across specialties. Ophthalmology is often encountered in referrals for diverse conditions, ranging from routine diagnostic assistance to urgent management of ocular emergencies [1]. However, the pattern, reasons, and outcomes of such referrals, especially in tertiary care settings, have not been comprehensively studied. This gap limits our understanding of how referrals impact clinical workflow and postgraduate (PG) training.

As primary responders to referral calls, PG residents face the dual challenge of managing diverse clinical presentations while simultaneously acquiring the skills to assess and manage cases independently [2]. Referrals offer a unique learning opportunity, fostering the development of diagnostic acumen, procedural competency, and interdisciplinary communication skills. Although referral cases contribute to the residents' training, their progressive learning over time has been sparsely documented.

Existing literature primarily focuses on specific clinical conditions or departmental workflows, often neglecting the comprehensive evaluation of referral patterns and their broader educational implications [3,4]. Furthermore, discrepancies in the accuracy of initial assessments by PG residents and senior ophthalmologists or consultants are not well-explored, leaving a lacuna in understanding the dynamics of training efficacy.

The study aims to bridge these gaps by analyzing the pattern, reasons, and timing of ophthalmology referrals in a tertiary care hospital. It also evaluates the impact of referral on the learning curve of PG residents with a focus on their ability to handle cases independently and accurately over time. By addressing these objectives, the study seeks to provide actionable insights to optimize both patient care and the training of future ophthalmologists.

## Materials and methods

A hospital-based, cross-sectional, observational study was performed at a tertiary care hospital in central India over 18 months. The study involved interdepartmental referrals to the ophthalmology department of the hospital. The study adhered to the tenets of the Declaration of Helsinki for biomedical research. Ethical approval was obtained from the Institutional Human Ethics Committee (Letter of Permission No. 2020/PG/July/29, granted on 24/02/2021).

The study was conducted at the All India Institute of Medical Sciences, Bhopal, Madhya Pradesh, India, from April 2021 to October 2022. A total of 1061 referrals were received and analyzed during the study period. Although there was no previous data on referral trends in this setting, the sample size was sufficient to capture referral trends and PG residents' training influence extensively, according to hospital records and annual patient load statistics.

All referrals to the ophthalmology department seen over the course of the study period by the second-year PG students were included. Referrals were identified through the hospital’s referral records. The data collected encompassed all referral calls, regardless of the referring department, time of day, or patient demographics. Cases with incomplete referral details were excluded.

Data collection

Data were collected using a structured pre-designed form (Figure 3 of Appendices). The form provided information regarding patient demographics (gender and age), referral departments, systemic diagnosis, reasons for referral, timing of referral calls (morning: 8 am-4 pm, evening: 4 pm-12 am, and night: 12 am-8 am), and ocular findings.

The referral calls were classified into two primary categories: Category A and Category B. Category A classification was based on the type of consultation requested and included four subtypes. Conservative referrals involved calls made to assess the ocular manifestations of systemic diseases, such as diabetic or hypertensive retinopathy. Interventional referrals were those seeking medical or surgical ophthalmic intervention for active conditions like ocular emergencies or trauma. Pre-procedural referrals were made for obtaining ophthalmology clearance prior to systemic procedures such as lumbar puncture or electroconvulsive therapy. Aid in diagnosis referrals were aimed at identifying ocular signs that could assist in diagnosing systemic illnesses, such as papilledema in cases of raised intracranial pressure or idiopathic intracranial hypertension, and the presence of a Kayser-Fleischer (KF) ring in Wilson’s disease. On the other hand, Category B classification was based on the timing of the referral call. Calls received between 8:00 am and 4:00 pm were considered day referrals, those received between 4:00 pm and 10:00 pm were categorized as evening referrals, and those received between 10:00 pm and 8:00 am were designated as night referrals.

PG resident competency was determined by a structured comparison of their findings and decision-making regarding management with those of senior resident doctors or consultants. The trends in discrepancy and independently handled referrals were tracked every six months over the study period to assess the learning curve of the PG residents. The primary outcome was a reduction in resident-to-consultant discrepancy, and the secondary outcome was cases handled independently.

Statistical analysis

Data were collected and graphical representations were prepared using Microsoft Office Excel 2019 (Microsoft Corporation, Redmond, WA). Statistical analysis was conducted using IBM SPSS Statistics for Windows, Version 23.0 (IBM Corp., Armonk, NY, USA). Categorical variables were expressed as frequencies and percentages, while continuous variables were summarized using mean and standard deviation. The association between categorical variables was assessed using the Chi-square test or Fisher’s exact test, as appropriate. A p-value of <0.05 was considered statistically significant.

## Results

A total of 1061 ophthalmology referrals were analyzed during the study period. The mean age of referred patients was 39.76±22.26 years, ranging from four months to 92 years, with a male predominance (n=650, 61.26%). The majority of referrals were received on weekdays (n=782, 73.7%), predominantly during the morning hours (8:00 am to 4:00 pm), accounting for 672 referrals (63.34%), while the fewest referrals were received at night (n=47, 4.43%) (Table 1).

**Table 1:** Distribution of patients according to age, gender, weekdays, and timing of referral calls

Age groups (years)	N=1061	%
Pediatric (<18 years)	203	19.13
Young adult (18-40)	346	32.61
Middle-aged (41-60)	296	27.89
Elderly (>60)	216	20.35
Gender		
Male	650	61.26
Female	411	38.74
According to weekdays		
Weekdays	782	73.70
Weekends	279	26.30
Time of referral call		
8 am-4 pm	672	63.34
4 pm-10 pm	342	32.23
10 pm-8 am	47	4.43

The major indications for referral included aid in diagnosis in 333 cases (31.39%), conservative management in 285 cases (26.86%), pre-procedural assessments in 189 cases (17.81%), and interventional management in 251 cases (23.66%). These findings are summarized in Table 2. 

**Table 2:** Distribution of referral calls according to the type of consultation required

Reason for consultation required	N=1061	%
Conservative management	285	26.86
Interventional management	251	23.66
Pre-procedural assessment	189	17.81
Aid in diagnosis	333	31.39
Conservative + pre-procedural	1	0.09
Conservative + aid in diagnosis	1	0.09
Pre-procedural assessment + aid in diagnosis	1	0.09

**Figure 1:**
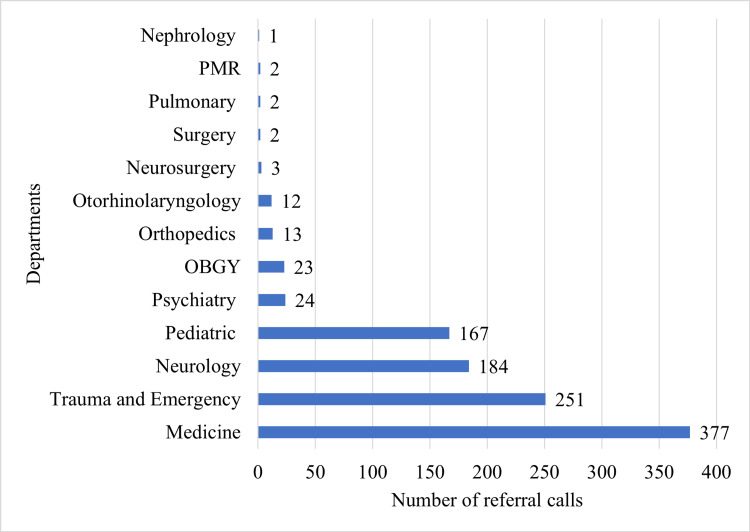
Bar diagram showing the distribution of referrals across various departments OBGY, obstetrics and gynecology; PMR, physical medicine and rehabilitation

The highest number of referrals originated from the medicine department (n=377, 35.53%) and the trauma and emergency department (n=251, 23.66%), followed by referrals from the neurology and pediatrics departments. The distribution of referrals across various departments is illustrated in Figure 1.

The analysis showed that aid in diagnosis was the most common referral reason in pediatrics (48.3%), interventional management was highest in young adults (32.1%), while conservative management predominated in the elderly (51.4%). Rare mixed categories (≤0.3%) were also observed. The association between age group and reason for consultation was statistically significant (p<0.001) (Table 3).

**Table 3:** Association of age group with reason for consultation (N=1061)

Reason for consultation	Pediatrics (<18)	Young adult (18-40)	Middle-aged (41-60)	Elderly (>60)	Total	p-value
Conservative management	8 (3.9%)	60 (17.3%)	106 (35.8%)	111 (51.4%)	285	<0.001
Interventional management	44 (21.7%)	111 (32.1%)	58 (19.6%)	38 (17.6%)	251	
Pre-procedural assessment	52 (25.6%)	75 (21.7%)	45 (15.2%)	17 (7.9%)	189	
Aid in diagnosis	98 (48.3%)	100 (28.9%)	85 (28.7%)	50 (23.1%)	333	
Conservative + pre-procedural	0 (0.0%)	0 (0.0%)	1 (0.3%)	0 (0.0%)	1	
Conservative + aid in diagnosis	0 (0.0%)	0 (0.0%)	1 (0.3%)	0 (0.0%)	1	
Pre-procedural assessment + aid in diagnosis	1 (0.5%)	0 (0.0%)	0 (0.0%)	0 (0.0%)	1	
Total	203	346	296	216	1061	

There were no ocular findings in 648 of the referred cases (61.07%), with the most frequent abnormal findings being ocular trauma in 148 cases (13.95%), diabetic retinopathy in 83 cases (7.83%), and papilledema in 53 cases (4.99%) (Table 4).

**Table 4:** Pattern of ocular findings in referral calls KF, Kayser-Fleischer

Ocular findings	N (1061)	%
No ocular findings	648	61.07
Ocular trauma	148	13.95
Diabetic retinopathy	83	7.83
Papilledema	53	4.99
Hypertensive retinopathy	21	1.97
Chemical burn	15	1.41
Cranial nerve palsy	15	1.41
Lens-induced glaucoma	14	1.31
Proptosis	12	1.13
Optic atrophy	12	1.13
Mixed diabetic and hypertensive retinopathy	11	1.03
KF ring	9	0.84
Retinal detachment	9	0.84
Optic neuritis	4	0.37
Retinal vascular occlusions	4	0.37
Exposure keratopathy	3	0.28

The analysis demonstrates that trauma was more common in younger age groups (15% in paediatrics and 19% in young adults), while retinopathy (diabetic and hypertensive) predominated in the elderly (31%), with significant differences across categories (p<0.001) (Table 5).

**Table 5:** Pattern of ocular findings among different age groups

Ocular finding (N=1061)	Pediatrics (<18)	Young adult (18-40)	Middle-aged (41-60)	Elderly (>60)	Total	p-value
Normal	145 (71%)	223 (64%)	189 (64%)	91 (42%)	648	<0.001
Trauma	30 (15%)	66 (19%)	32 (11%)	20 (9%)	148	
Retinopathy	0 (0%)	8 (2%)	29 (10%)	67 (31%)	104	
Papilledema	8 (4%)	20 (6%)	13 (4%)	12 (6%)	53	
Other	20 (10%)	29 (8%)	33 (11%)	26 (12%)	108	
Total	203	346	296	216	1061	

Of the total referrals, 251 cases required specific interventions (23.66 %), with medical management in 135 cases (53.78%) and surgical procedures in 116 cases (46.21%), as shown in Table 6. Among patients requiring surgical procedures were the repair of traumatic ocular injuries, cataract surgeries, and other emergency interventions.

**Table 6:** Distribution of referral calls according to ocular intervention *No statistically significant difference between medical and surgical interventions (p>0.05) Chi-square test for independence was used to calculate the p-value.

Interventions	N (251)	%	P-value
Medical intervention	135	53.78	≈0.18*
Surgical intervention	116	46.21	

Over the course of the study, a significant improvement in the clinical competency of second-year PG residents was observed. The proportion of referrals that were managed by residents with findings correlating accurately with those of senior residents or consultants with few changes increased from 221 cases (62.96%) at the beginning of the study to 312 cases (91.22%) by the end of the 18-month period. Correspondingly, the discrepancy rate in clinical findings between PG residents and their senior counterparts decreased markedly, from 130 cases (37.01%) to 30 cases (8.77%), indicating a substantial improvement in both diagnostic accuracy and clinical decision-making skills. These trends are detailed in Table 7.

**Table 7:** Six-monthly learning pattern analysis of PG trainee residents *This is highly statistically significant (p<0.001), indicating a strong association between the time period and improvement in diagnostic concordance with seniors' findings. It supports the conclusion that PG residents significantly improved their performance over time. Chi-square test for trends was used to calculate the p-value. PG, postgraduate

Parameters	First six months (n=351)	Second six months (n=368)	Third six months (n=342)	p-value
Referrals managed by the PG showed ocular examination findings and management that correlated with the senior resident’s/consultant’s findings, with few changes	221 (62.96%)	280 (76.08%)	312 (91.22%)	<0.0001*
Discrepancy in clinical findings and management in referrals between postgraduates and their senior residents/consultants	130 (37.01%)	88 (23.91%)	30 (8.77%)	

**Figure 2:**
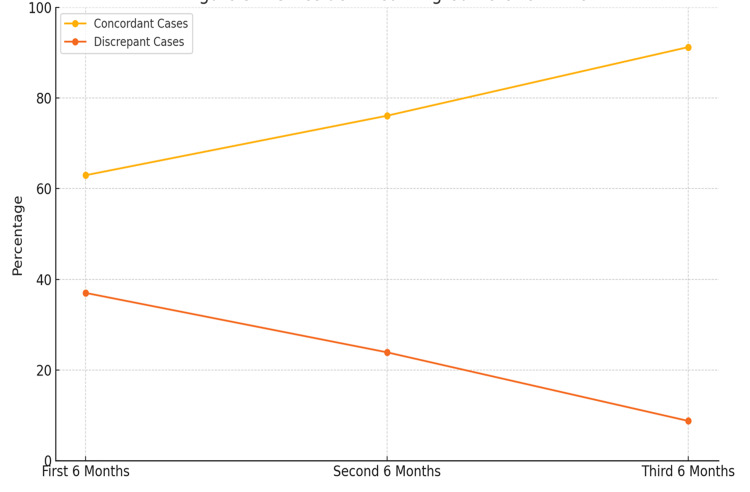
PG resident learning curve over time PG, postgraduate

**Figure 3:**
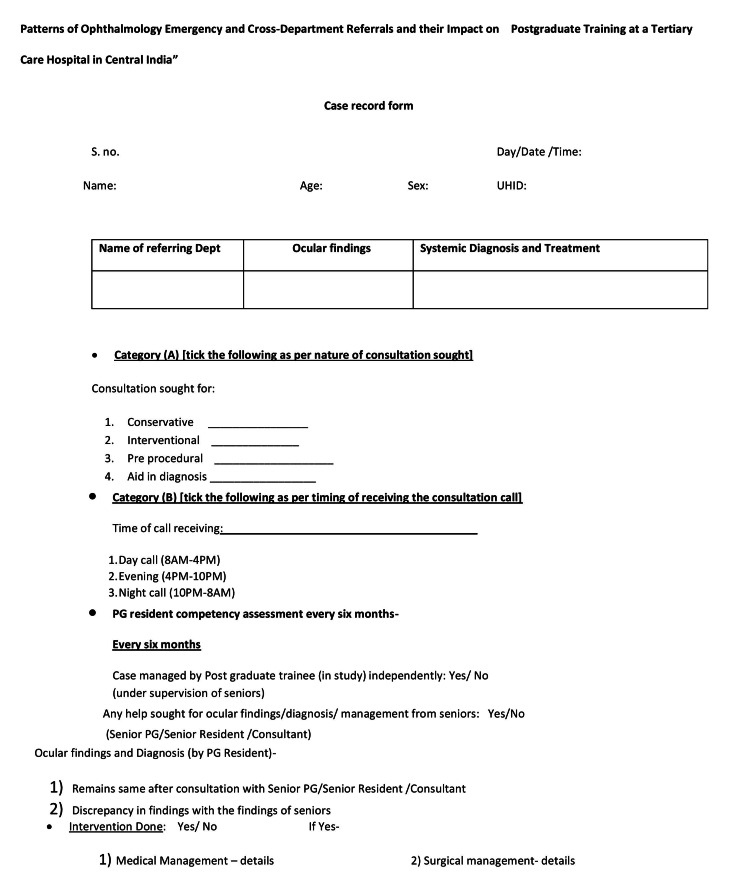
Case proforma developed by authors

Diagnostic concordance with seniors improved significantly over the 18-month period, indicating effective experiential learning, with targeted teaching and supervised feedback (Figure 2).

The study emphasized the gradual improvement of PG residents' skills through systematic exposure to referral cases. A structured approach, targeted teaching and training sessions, and supervised feedback played an important role in enhancing diagnostic and management skills, and the role of hands-on clinical experience was particularly highlighted in residency training.

The analysis showed that agreement between residents and the seniors varied significantly across ocular findings (p<0.001). Normal cases were most accurate (95% independent), while discrepancies were higher in trauma (26%), diabetic and hypertensive retinopathy (28%), and particularly in “other” cases (47%); identifying these as key areas, targeted resident teaching and training sessions were done (Table 8).

**Table 8:** Ocular findings and agreement with seniors

Ocular findings	Independently managed	Correlated with changes	No correlation	Total	p-value
Normal	615 (95%)	33 (5%)	0 (0%)	648	<0.001
Trauma	110 (74%)	35 (24%)	3 (2%)	148	
Diabetic and hypertensive retinopathy	75 (72%)	28 (27%)	1 (1%)	104	
Papilledema	43 (81%)	10 (19%)	0 (0%)	53	
Other	57 (53%)	48 (44%)	3 (3%)	108	
Total	900 (85%)	154 (15%)	7 (1%)	1061	

Table 9 presents the results of a binary logistic regression assessing the association between age group and abnormal ocular findings. Elderly patients (>60 years) were used as the reference category. The table shows the logistic regression coefficient (B), odds ratio (OR=Exp(B)), 95% CI for the OR, and p-values for each age group compared to the elderly.

**Table 9:** Binary logistic regression showing the association between age group and abnormal ocular findings Elderly (>60 years) group was the reference category. B, regression coefficient

Age group (ref=elderly >60)	B	OR (Exp(B))	95% CI for OR	p-value
Pediatrics (<18)	-1.234	0.29	0.19-0.44	<0.001
Young adult (18-40)	-0.912	0.4	0.28-0.57	<0.001
Middle-aged (41-60)	-0.886	0.41	0.29-0.59	<0.001
Elderly (>60)	Reference	1	-	-

The analysis demonstrated that age group was a significant predictor of abnormal ocular findings (χ²=42.65, df=3, p<0.001). Compared with elderly patients, pediatric participants (<18 years) had 71% lower odds of abnormal ocular findings (OR=0.29, 95% CI 0.19-0.44, p<0.001). Young adults (18-40 years) had 60% lower odds (OR=0.40, 95% CI 0.28-0.57, p<0.001), and middle-aged adults (41-60 years) had 59% lower odds (OR=0.41, 95% CI 0.29-0.59, p<0.001). Thus, elderly patients consistently showed the highest likelihood of abnormal ocular findings compared to all younger age groups.

## Discussion

Ophthalmology consultation requests are an integral component of multidisciplinary care in tertiary hospitals, especially dealing with ocular problems that need specialist input and where systemic diseases frequently manifest with ocular signs [5]. These referrals are critical in early detection, diagnosis, and timely management [6]. They also play a significant role in the learning process and skill acquisition of PG residents, as handling these calls is an integral part of their training [7].

This prospective, observational study, among the first of its kind in India, adds significantly to the limited national literature by systematically evaluating the nature and patterns of interdepartmental ophthalmology referrals and their educational value for PG training. By assessing 1061 referrals over 18 months, we have captured key insights into referral practices, diagnostic outcomes, workload distribution, and competency development of PG residents.

Referral trends by demographics

In this study, ophthalmology referrals encompassed a broad age spectrum, ranging from infants to elderly patients, with the highest referral frequencies observed in the 18-40 and 41-60 year age groups. Males constituted 650 cases (61.26%), resulting in a male-to-female ratio of 1.58, a demographic pattern comparable to findings reported by Jafari et al. and Nikandish et al. [8,9]. This gender disparity likely reflects the increased burden of ocular conditions among adult males, potentially attributed to occupational hazards and lifestyle-related factors.

Timing and workload implications

The temporal distribution of referral calls demonstrated a clear peak during weekdays, with Fridays accounting for the highest number of referrals (n=169, 15.93%), and relatively fewer referrals received on Sundays (n=123, 11.59%) and holidays (n=42, 3.96%). This trend aligns with observations by McDonald et al. and Khathlan et al., who similarly reported increased referral activity during routine inpatient rounds on weekdays [7,10].

Furthermore, the majority of referrals occurred during daytime hours (8:00 am-4:00 pm), while nighttime referrals (10:00 pm-8:00 am) comprised only 47 cases (4.43%). The skewed timing may lead to disproportionate workloads during outpatient clinic hours, potentially disrupting scheduled patient care. While night-time referrals were fewer, they often involved serious emergencies, requiring round-the-clock ophthalmology resident coverage. Given this, hospitals must consider these patterns when designing emergency duty rosters and triage protocols to ensure both quality patient care and optimal training opportunities for residents.

Referral patterns

Diagnostic assistance emerged as the leading indication for referral, accounting for 333 cases (31.39%), followed by conservative management in 285 cases (26.86%), and interventional management in 251 cases (23.66%). Additionally, pre-procedural clearance was the reason for 189 referrals (17.81%), offering further insights into the diverse patterns of ophthalmology consultations analyzed in this study. These findings are consistent with those reported by Channa et al. and Galvis et al. [11,12], underscoring the central role of ophthalmologists in a multidisciplinary hospital setting, particularly in the evaluation of ocular signs of systemic disease.

Referral appropriateness and overuse

Analysis of the ocular findings revealed that a majority of referrals showed no significant pathology, with 648 cases (61.07%) being clinically unremarkable. This high proportion of normal findings may suggest a tendency toward overutilizing ophthalmology services. Several factors may underlie this, including a low threshold for referral, limited ophthalmic examination skills among non-ophthalmology departments, and a defensive medical culture prioritizing comprehensive documentation. Similar trends have been observed internationally; Jafari et al. reported that many emergency ophthalmology consultations yielded non-urgent or normal findings in a referral hospital setting in Iran [8]. Likewise, Galvis et al. found that a significant proportion of ophthalmic emergency consultations in Colombia were either nonspecific or revealed no pathology [12].

Referral trends by departments

The departments generating the highest number of referrals were medicine (n=377, 35.53%) and trauma and emergency (n=251, 23.66%), together accounting for 628 referrals (59.19%) of the total. These findings are in alignment with those reported by Joshi and Nari et al. [3,13], where the medicine and emergency departments similarly contributed substantially to ophthalmology consultations. This trend highlights the critical role of ophthalmologists in managing systemic diseases with ocular manifestations and providing prompt care for acute ophthalmic emergencies.

The most frequent abnormal ocular findings were trauma (n=148,13.95%), diabetic retinopathy (n=83,7.83%), and papilledema (n=53,4.99%). These patterns align with earlier studies from India and Latin America [3,12], highlighting the importance of early detection of systemic complications through ocular examination.

Educational value and competency mapping

A key strength of this study is the longitudinal tracking of PG resident performance over 18 months. We observed a significant improvement in diagnostic accuracy and decision-making, with concordance between residents’ and consultants’ findings rising from 221 referrals (62.96%) to 312 referrals (91.22%). Simultaneously, the rate of discrepancy in ocular findings and management decreased from 130 referrals (37.01% ) to 30 referrals (8.77%). These outcomes align with the principles of the Competency-Based Medical Education (CBME) framework implemented by the National Medical Commission (NMC) in India, and the AETCOM module that emphasizes attitude, ethics, and communication [14].

Regular exposure to referral cases enhances multiple core competencies, including clinical acumen, interdisciplinary communication, clinical reasoning under supervision, and recognizing systemic-ocular correlations. Studies from Western settings support this; Noble et al. emphasized the gaps in ophthalmic training in undergraduate medical curricula in Canada and advocated for hands-on residency training to bridge this gap [15]. Deaner et al. similarly emphasized the value of structured clinical exposure to improve diagnostic accuracy in eye emergencies [16].

Global perspective and triage practices

Findings from our study resonate with global data. Heiferman et al. and McDonald et al. reported that many ophthalmology consultations from emergency departments in the United States and Canada, respectively, were either non-urgent or stemmed from uncertainty among non-ophthalmic physicians [2,7]. A key distinction, however, is that many Western institutions employ structured triage systems to optimize referral flow. The absence of such mechanisms in most Indian tertiary hospitals may contribute to inefficiencies and missed educational opportunities. Formal triage and decision-support systems could enhance patient care and resident training.

Recommendations for practice and training reform

Based on our findings, we propose several evidence-based, cost-effective interventions to improve the quality of ophthalmology referrals and enhance PG training, in alignment with the NMC curriculum. Incorporating structured referral checklists into electronic medical records can ensure consistency and reduce unnecessary consultations. A senior resident or consultant-led triage system during peak hours can help prioritize emergencies and streamline workload. Cross-disciplinary orientation through basic eye screening training for non-ophthalmology departments can boost confidence and reduce over-referrals. Mobile decision aids, such as apps or printed flowcharts, can assist junior residents in managing ophthalmic emergencies during off-hours. Additionally, integrating referral audits and reflective learning tools into resident assessments or logbooks can promote clinical reasoning and continuous self-improvement. These interventions are practical, easy to implement, and hold the potential to significantly strengthen both patient care and clinical training.

Strengths and limitations

One of the key strengths of this study lies in its comprehensive and first-of-its-kind evaluation of interdepartmental ophthalmology referral patterns in India, providing valuable insight into real-world clinical workflows within a tertiary care setting. With a robust sample size of 1061 prospectively collected referrals and a longitudinal follow-up over 18 months, the study was able to capture evolving trends in referral practices and their direct educational impact on PG training. The structured categorization of referrals and interventions allowed for detailed analysis, while the tracking of PG residents' competency demonstrated a clear and significant improvement in diagnostic accuracy and decision-making over time, underscoring the value of experiential learning within the CBME framework. This study uniquely highlights how routine referral management contributes meaningfully to PG education by offering practical, hands-on exposure across diverse clinical scenarios.

However, several limitations must be acknowledged. Being a single-center study, its generalizability to other institutions may be limited, as referral practices and training structures can vary widely across settings. Importantly, while the reduction in diagnostic discrepancies is encouraging, the study did not delve deeply into the types of errors or specific areas of difficulty faced by trainees. Finally, institutional protocols and departmental cultures may have influenced referral behavior, limiting the external applicability of findings. Future multicentric studies incorporating qualitative feedback and outcome-based metrics would be valuable to build on these findings and develop standardized models for referral training in ophthalmology residency programs.

Recommendations and future directions

This study underscores the importance of adopting a structured approach to managing ophthalmology referrals, notably to support and enhance PG training. Interventions like targeted teaching modules, real-time feedback systems, and standardized referral protocols can significantly improve residents' diagnostic skills and clinical confidence. These strategies not only benefit medical education but also elevate the quality of patient care. Future multicentric studies should aim to evaluate the effectiveness of these interventions across diverse settings to validate outcomes and facilitate the development of standardized, scalable referral training models.

## Conclusions

Emergency and interdepartmental ophthalmology referrals serve a dual purpose: they are pivotal for timely patient care and represent a rich, practical learning platform for PG residents. Actively involving trainees in structured referral management processes helps strengthen their clinical skills and competencies while ensuring accurate and efficient care. As this study shows, consistent supervision, targeted teaching and training sessions, feedback, and experiential learning may help narrow diagnostic gaps. Strengthening ophthalmology training through streamlined protocols and interdisciplinary collaboration holds great potential to improve outcomes for both patients and future ophthalmologists.

## References

[1] Prudhomme N, Kwok ES, Olejnik L, White S, Thiruganasambandamoorthy V (2019). A health records review of outpatient referrals from the emergency department. Emerg Med Int.

[2] Heiferman MJ, Khanna S, Gu D, Agron S, Eichinger SE, Bryar PJ (2019). Emergency department ophthalmology consultations in a tertiary care hospital. J Acad Ophthalmol.

[3] Joshi RS (2011). Study of referral pattern to ophthalmology outpatient department from various departments in the medical college. J Indian Med Assoc.

[4] Schachat AP, McDonnell PJ, Petty BG (1989). Ophthalmology consultations at a large teaching hospital. Metab Pediatr Syst Ophthalmol.

[5] Alabbasi OM, Al-Barry M, Albasri RF (2017). Patterns of ophthalmic emergencies presenting to a referral hospital in Medina City, Saudi Arabia. Saudi J Ophthalmol.

[6] Samoilă O, Ostriceanu S, Samoilă L (2016). Epidemiology of ocular emergencies in Cluj ophthalmology clinic. Rom J Ophthalmol.

[7] McDonald HM, Iordanous Y (2022). Ophthalmology on call: evaluating the volume, urgency, and type of pages received at a tertiary care center. Cureus.

[8] Jafari AK, Bozorgui S, Shahverdi N, Ameri A, Akbari MR, Salmasian H (2012). Different causes of referral to ophthalmology emergency room. J Emerg Trauma Shock.

[9] Nikandish M (2020). Patterns of the eye emergencies referring to the Valiasr hospital in Birjand, Iran, during the "Nowruz" Holiday 2019. Mod Care J.

[10] Khathlan AA (2021). Community ophthalmology clinic utilization and morbidities results from a private primary healthcare center in Saudi Arabia. Saudi J Ophthalmol.

[11] Channa R, Zafar SN, Canner JK, Haring RS, Schneider EB, Friedman DS (2016). Epidemiology of eye-related emergency department visits. JAMA Ophthalmol.

[12] Galvis V, Díaz AL, Ochoa ME, Rey JJ, Ardila LC, Olivero LP, Tello A (2019). Primary causes of emergency ophthalmological consultations at a tertiary care institution in Colombia. MedUNAB.

[13] Nari J, Allen LH, Bursztyn LL (2017). Accuracy of referral diagnosis to an emergency eye clinic. Can J Ophthalmol.

[14] Sharma R, Bakshi H, Kumar P (2019). Competency-based undergraduate curriculum: a critical view. Indian J Community Med.

[15] Noble J, Somal K, Gill HS, Lam WC (2009). An analysis of undergraduate ophthalmology training in Canada. Can J Ophthalmol.

[16] Deaner JD, Amarasekera DC, Ozzello DJ (2021). Accuracy of referral and phone-triage diagnoses in an eye emergency department. Ophthalmology.

